# Binding Mode Analysis of Zerumbone to Key Signal Proteins in the Tumor Necrosis Factor Pathway

**DOI:** 10.3390/ijms16022747

**Published:** 2015-01-26

**Authors:** Ayesha Fatima, Ahmad Bustamam Hj. Abdul, Rasedee Abdullah, Roghayeh Abedi Karjiban, Vannajan Sanghiran Lee

**Affiliations:** 1 UPM-MAKNA Cancer Research Laboratory, Institute of Biosciences, University Putra Malaysia, 43400 Serdang, Malaysia; E-Mails: ahmadbstmm@yahoo.com (A.B.H.A.); rasedee@gmail.com (R.A.); 2 Faculty of Pharmaceutical Sciences, UCSI University, 1-Jalan Menara Gading, Taman Connaught, Cheras, 56000 Kuala Lumpur, Malaysia; 3 Department of Microbiology and Pathology, Faculty of Veterinary Medicine, University Putra Malaysia, 43400 Serdang, Malaysia; 4 Department of Chemistry, Faculty of Science, University Putra Malaysia, 43400 Serdang, Malaysia; E-Mail: rosa.abedi@gmail.com; 5 Enzyme and Microbial Technology Research Centre, Faculty of Biotechnology and Biomolecular Sciences, Universiti Putra Malaysia, 43400 Selangor, Malaysia; 6 Department of Chemistry, Faculty of Science, University Malaya, Petaling Jaya, 50603 Selangor, Malaysia

**Keywords:** zerumbone, Inhibitor of κ-B kinase (IKK), nuclear factor κ-light-chain-enhancer of activated B cells (NF-κB), CHARMm based docking software (CDOCKER), molecular docking

## Abstract

Breast cancer is the second most common cancer among women worldwide. Several signaling pathways have been implicated as causative and progression agents. The tumor necrosis factor (TNF) α protein plays a dual role in promoting and inhibiting cancer depending largely on the pathway initiated by the binding of the protein to its receptor. Zerumbone, an active constituent of *Zingiber zerumbet*, Smith, is known to act on the tumor necrosis factor pathway upregulating tumour necrosis factor related apoptosis inducing ligand (TRAIL) death receptors and inducing apoptosis in cancer cells. Zerumbone is a sesquiterpene that is able to penetrate into the hydrophobic pockets of proteins to exert its inhibiting activity with several proteins. We found a good binding with the tumor necrosis factor, kinase κB (IKKβ) and the Nuclear factor κB (NF-κB) component proteins along the TNF pathway. Our results suggest that zerumbone can exert its apoptotic activities by inhibiting the cytoplasmic proteins. It inhibits the IKKβ kinase that activates the NF-κB and also binds to the NF-κB complex in the TNF pathway. Blocking both proteins can lead to inhibition of cell proliferating proteins to be downregulated and possibly ultimate induction of apoptosis.

## 1. Introduction

Cancer is one of the leading causes of deaths worldwide [1]. In Malaysia, lung cancer (16.3%) and breast cancer (32.1%) account for the leading causes of death among males and females, respectively [2]. Several available options of anticancer drugs are non-selective and toxic. This evident void generates the need for finding newer and safer therapies. The use of alternate medicine has always been an interesting area in medical explorations. Several studies have reviewed the use of herbal drugs alone and in combination with chemotherapy to inhibit the progression of the disease [3,4,5,6,7,8,9].

**Figure 1:**
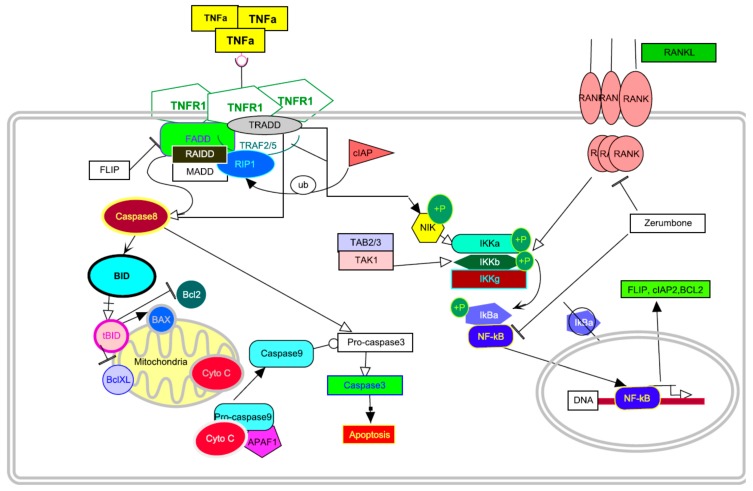
Reported action of zerumbone against against nuclear factor κ-light-chain-enhancer of activated B cells (NF-κB) and κ-B ligand (RANKL).

Zerumbone is a sesquiterpene obtained from *Zingiber zerumbet* Smith. It is known to exhibit anti-cancer activity against several cancers by modulating several proteins to induce apoptosis [10]. Several articles have identified key proteins that can be inhibited by zerumbone for arresting cancer cell growth [11,12,13,14,15,16,17,18,19]. Prasannan *et al.* [19] reviewed key pathways such as tumor necrosis factor signaling pathway and the phosphinositide-3-kinase/Akt/mTOR pathway modulated by zerumbone. It has shown anti-inflammatory and chemopreventive activity against colon and skin cancer [14,20,21]. Reports have also been published on the apoptotic activity of zerumbone on liver, ovary and cervix as well as leukemia [11,12,13,16,18]. It has been reported to act as a modulator of osteoclastogenesis induced by receptor activated NF-κB ligand (RANKL) and breast cancer [22]. Figure 1 shows the effect of zerumbone on the TNF pathway and RANKL.

Although zerumbone has been studied extensively in the laboratory, but determining the precise binding target and the molecular level events that may occur between the drug and target has not fully understood. Some researchers have reported that the apoptotic mechanism of zerumbone could be due to the formation of Michael adducts that it is an unsaturated carbonyl group forms with glutathione to remove it, which increases the intracellular redox potential of cancerous cell as compared to the normal cells, ultimately leading to apoptosis of the cancer cells [11,23]. Ligand protein interactions can be investigated using docking programs. Docking techniques are useful since they allow a better understanding of the molecular events happening at the binding interface of ligand-protein interaction site. Their utility is paramount in complementing and supplementing the experimentally determined data. Using CHARMm based docking software (CDOCKER) of the Discovery Studio 2.5.5 (Accelrys Inc., San Diego, CA, USA) suite of programs, docking studies were carried out to evaluate which proteins are the most likely targets of zerumbone and determine the exact binding mechanism of the molecule with its target protein [24,25]. CDOCKER applies grid-based molecular dynamics simulated annealing protocol by using CHARMM force field while devising the appropriate position of the ligand in the active pocket. The algorithm offers flexible ligand docking where the non-bonded interactions are softened during the docking procedure but removed during the final minimization process [25]. The protein was held rigid during the entire process.

In this study, we explored the binding of zerumbone to key molecules of the TNF signaling pathway. The pathway was selected because several experimentally determined studies have already been published to elucidate the importance of zerumbone as an inhibitor of cancer proliferating compound in this pathway. However, to the best of our knowledge, there is no published study of molecular events occurring in the pathway with zerumbone. This study explores the detailed binding modes of zerumbone with the target proteins.

## 2. Results and Discussion

### 2.1. CDOCKER Energy, CDOCKER Interaction Energy and the Binding Energy

**Figure 2:**
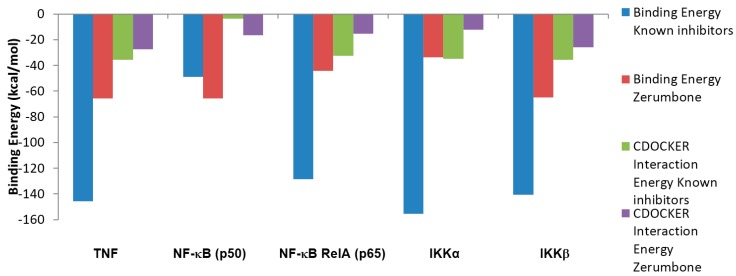
Energy profile of known inhibitor molecules and zerumbone from CHARMM based docking software (CDOCKER).

**Figure 3:**
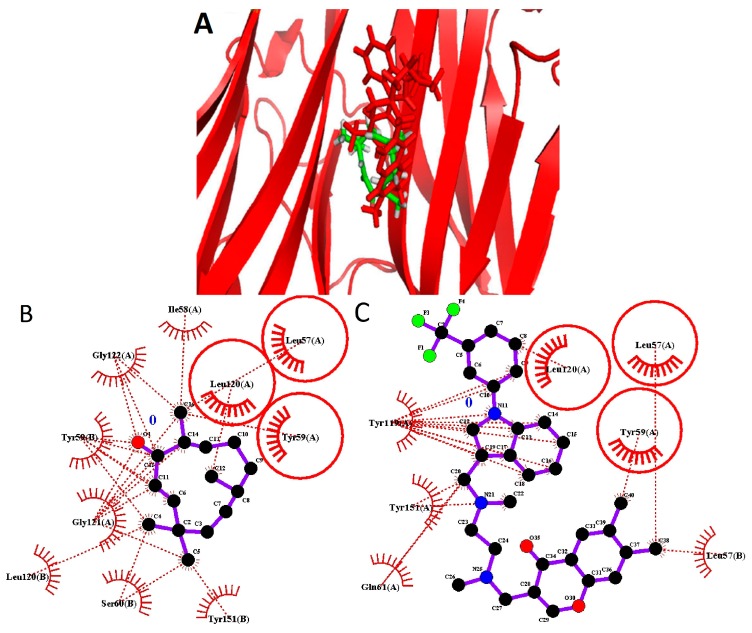
Zerumbone and the known inhibitor binding to Tumor Necrosis Factor α. (**A**) Zerumbone (green) binding to Tumor Necrosis Factor α is presented. The known ligand (red) and zerumbone bind into the same active pocket; (**B**) two dimensional protein-lilgand interactions plot (LIGPLOT) of zerumbone in the active site of the protein; (**C**) LIGPLOT of known inhibitor in the active site of the protein. The common residues are circled.

**Figure 4:**
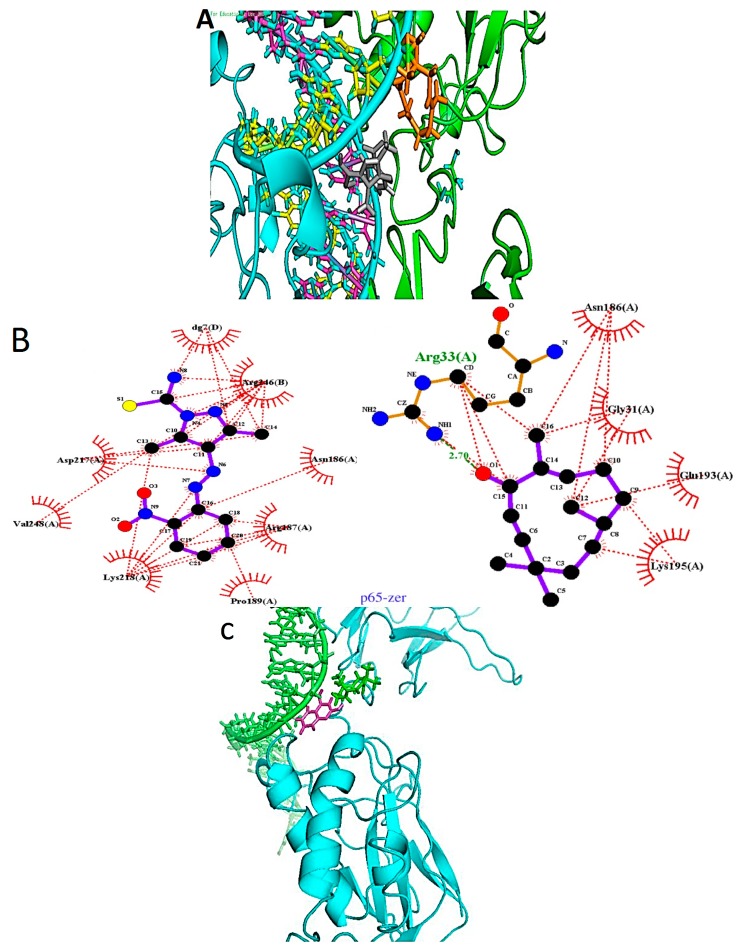

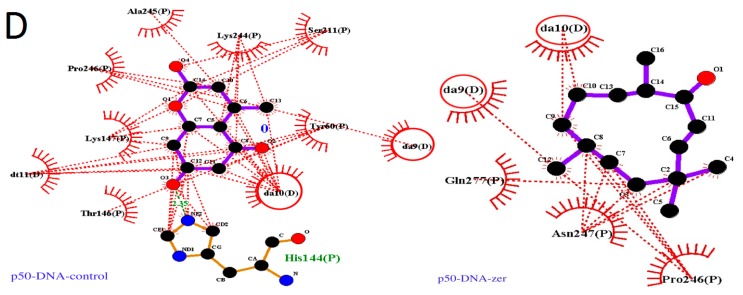
Binding of reported ligands and zerumbone to nuclear factor κB. (**A**) p65 component: Zerumbone (orange) is placed between the chain A (green) and the DNA ladder. The known ligand (grey) can be placed between the chain A and the major groove of the DNA. Chain B of the p65 is colored cyan; (**B**) The LIGPLOT image of the binding site showing interactions between known ligand and zerumbone with the pocket residues; (**C**) p50 component: Zerumbone (green) is placed between the p50 chain (cyan) and the DNA ladder (green). The known ligand (magenta) is embedded deeper in the major groove of the DNA; (**D**) The LIGPLOT image of the binding site showing interactions between known ligand and zerumbone with residues in the binding the pocket.

**Figure 5:**
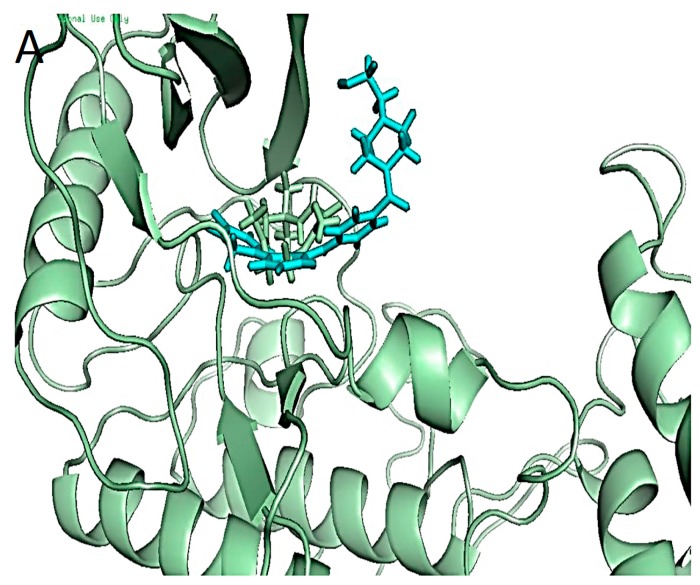

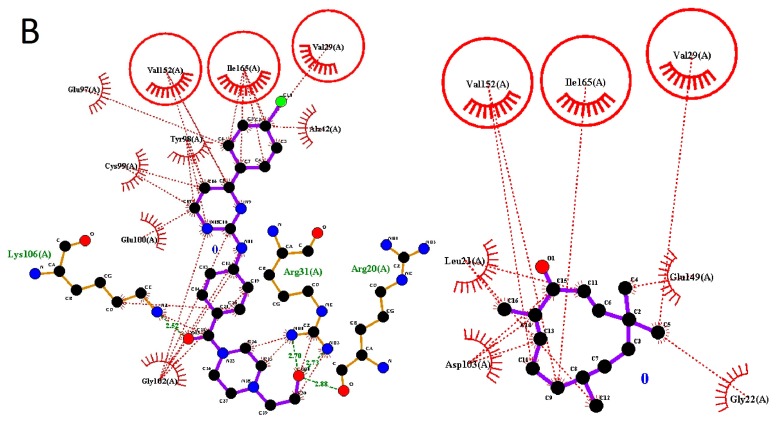
Binding of co-crystalized ligand and zerumbone with inhibitor of kappa-B kinase subunit beta (IKKβ). (**A**) Known ligand (cyan) and zerumbone (green) complexed with IKKβ; (**B**) The LIGPLOT results showing the interactions of zerumbone with binding pocket residues. The common interacting residues are circled.

**Figure 6:**
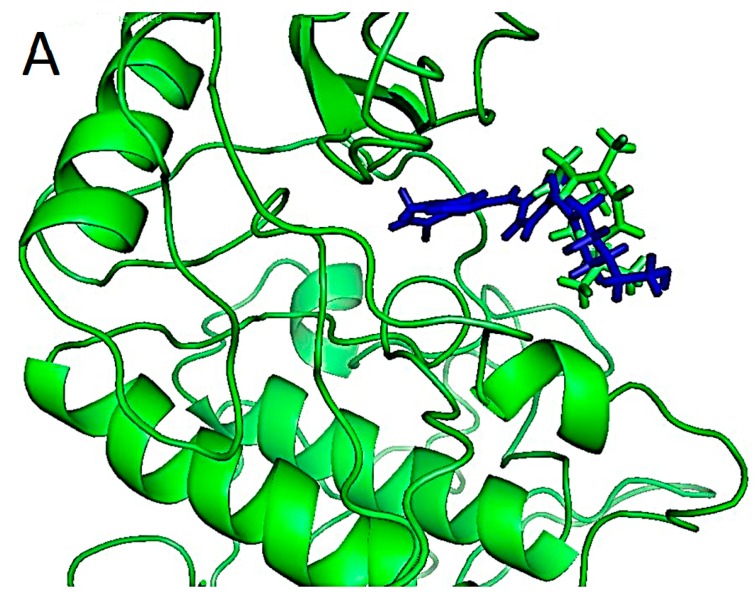

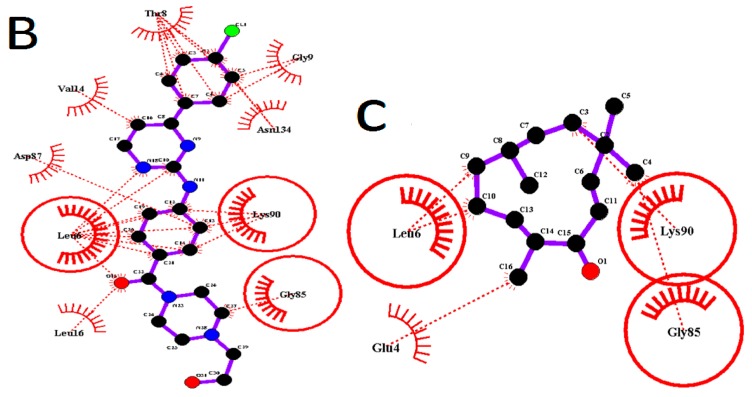
Known inhibitor and zerumbone docking to inhibitor of kappa-B kinase subunit alpha (IKKα). (**A**) Known inhibitor (blue) and zerumbone (green) bound in ATP pocket of the predicted human IKKα. Zerumbone hovers at the edge of the pocket, while the known ligand can be seen placed deeper into the pock; (**B**) The two dimensional interactions plot LIGPLOT result showing pocket residues for known inhibitor; (**C**) The LIGPLOT result showing pocket residues for zerumbone.

The values obtained for the binding energies and the CDOCKER interaction energy profiles of known inhibitor molecules either from the co-crystallized with target proteins or from the literature review as a control docking to compare the result with zerumbone to the proteins in TNF pathway molecules are presented in Figure 2. The compound structures in two dimensional interactions plot using a program for automatically plotting protein-ligand interactions (LIGPLOT) are shown in Figure 3, Figure 4, Figure 5 and Figure 6.

All results demonstrated a high binding affinity of the compounds with the negative binding energy and CDOCKER interaction energy. CDOCKER interaction energies results illustrated high negative values indicating the strong non-bonded interactions between zerumbone and the proteins from the van der Waals and the hydrophobic interactions. This is not surprising because zerumbone has a very rigid ring, has a high hydrophobicity and it does not have any rotatable bonds.

Binding energy was calculated for all docked structures with *in-situ* minimization of the ligand. Analysis of the binding energy showed that all reported inhibitor molecules had higher binding energies as compared with zerumbone. The results showed clearly that zerumbone had higher affinity towards cytoplasmic proteins such as NF-κB component p50 and IKKβ.

### 2.2. Binding Pocket Analysis

#### 2.2.1. Binding Pocket of the TNFα

TNF α signaling pathway is one of the major pathways in the promotion and proliferation of breast cancer cells. The PDB structure 2AZ5 presented by He *et al.* [26] is a homodimer co-crystalized with the inhibitor 6,7-Dimethyl-3-[methyl({1-[3-trifluoromethyl)phenyl)-1H-indol-3-yl]methyl]amino]ethyl]amino]methyl]-4H-chromen-4-one bound to two chains of TNFα. Figure 3 demonstrates zerumbone and the inhibitor binding to TNFα to the same active pocket of TNFα. The active pocket of the crystal structure is lined with Leu57, Ile58, Tyr59, Ser60, Gln61, Tyr119, Leu120, Gly121, Gly122, and Tyr151 of either chain. Zerumbone mainly binds in the same pocket with hydrophobic interactions with Leu 57, Tyr59, Gln61 and Tyr151, which are within 4 Å distance from the ligand. It is interesting to note that both chains contribute the same residues for interaction. However, no hydrogen boding between zerumbone and the chains has been detected. 

Rivas *et al.* [27] have reviewed the role of TNFα in breast cancer. According to Rivas *et al.* [27], breast cancer proliferates via the activation of p42/p44 MAPK, JNK, PI3-K/Akt pathways and transcriptional activation of nuclear factor-κ^®^ (NF-κB). Their experiments revealed that the blocking of tumour necrosis factor receptor 1 (TNFR1) or TNFR2 with specific antibodies at concentrations of 2–10 micromoles were able to impair the TNFα signaling as a breast tumor promoter. They also proposed that TNFα and NF-κB inhibition was essential for combating breast cancer [27]. Zerumbone is a well-studied molecule in this pathway. Researchers have already established that it targets several proteins along the pathway including nuclear factor κB (NF-κB) and inhibitor of κB kinase (IκB kinase or IKK), the main regulator of NF-κB [15].

TNFα binds to its receptor TNFR1 or TNFR2 as a homotrimer. He *et al.* [26] obtained a crystal structure of TNFα with 6,7-dimethyl-3-[(methyl{2-methyl({1-[3-(trifluoromethyl)phenyl]-1H-indol-3-yl}methyl)amino}ethyl)amino)methyl]-4H-chromen-4-one. Their results indicated that the inhibitor had IC_50_ of 22 micromoles, which was enough to stall TNFα activity. The binding of the co-crystalized inhibitor caused the dislocation of the third component of the trimer of TNF molecule thereby inhibiting TNF. Takada *et al.* [15] in their experiments showed that 25 micromoles of zerumbone was enough to suppress TNFα-induced activation of NF-κB in human lung carcinoma (H1299) cell lines.

Analyzing our results of binding poses of the inhibitor proposed by He *et al.* [26] and zerumbone, we observed that in the case of zerumbone, the molecule being smaller than the co-crystalized inhibitor appeared deeply buried within the homodimer pocket with the carbonyl group facing Tyr59 of one of the chains. The binding energy of the zerumbone-TNFα complex was −65.65 kcal/mol as compared with −145.6 kcal/mol of the co-crystallized inhibitor. To the best of our knowledge, no experimental data has been reported the zerumbone binding to TNFα. Hence, we could assume that sufficient affinity between the molecules could exist. However, since the available experimental evidence suggests that zerumbone enhanced TNF-induced cytotoxicity by suppression of the cytoplasmic proteins such as NF-κB [15] found downstream of the pathway and simultaneous induction of the pro-apoptotic proteins, such as caspases and death receptors [21], this result requires further investigation.

#### 2.2.2. Nuclear Factor κB Complex (NF-κB) Complex

The nuclear factor κB (NF-κB) is a group of transcriptional proteins that is held inhibited in the cytoplasm by its inhibitor IκBα [28,29,30,31,32]. Binding of the TNF α to its receptor TNFR1 triggers a signal that leads to the ubiquitination of the IκBα followed by translocation of NF-κB to the nucleus where it leads to the transcription of cell proliferating genes. The NF-κB complex is constitutively expressed in all tumor cells. The complex comprises NF-kB/Rel proteins (p50/p65) and the inhibitor kinase (IκBα). There is increasing evidence of its oncogenic role especially the RelA or p65 and the p50 components. Hence they become interesting targets for inhibition. Two recent studies have identified inhibitors that specifically target the two proteins [33,34]. Using docking studies and confirmatory biochemical assays, Law *et al.* [34] revealed that inhibiting serine276 phosphorylation of p65 (RelA) subunit can prevent angiogenesis and metastasis of several tumors. Nithya *et al.* [33] targeted the p50 subunit with several pharmacophores of withanolides and showed the possibility of inhibiting the DNA binding site targeted by NF-κB. An earlier study of inhibiting NF-κB by zerumbone published by Takada *et al.* [15] had pointed out that the constitutive expression of the protein can be blocked by zerumbone. They found that zerumbone had no direct effect on the NF-κB inhibition but it can inhibit TNF induced activation in human lung carcinoma cell lines (H1299) pre-treated with zerumbone. They suggested zerumbone strongly bound to the p65 component (RelA) of the complex. However specific details of binding were not presented. 

We used two structures with the individual components p50 and p65 bound to DNA for the docking studies. Unfortunately, a co-crystalized structure for the components with an inhibitor has not been solved to date. The p50-DNA (PDB ID: 1SVC) and p65-DNA (PDB ID: 2RAM) homodimer complexes were downloaded from the PDB database. Control docking was carried out with 5,7-dihydroxy-4-methylcoumarin for p50-DNA complex because of its reported activity against the complex [35] and with 3,5-dimethyl-4-[(2-nitrophenyl)diazenyl]pyrazole-1-carbothioamide for the p65-DNA complex reported by Law *et al*. [34].

We used the binding pockets proposed by the above authors and also confirmed them as reported elsewhere [34,35]. Our results of binding modes of the control docking and zerumbone are presented in Figure 5a,b. 3,5-Dimethyl-4-[(2-nitrophenyl)diazenyl]pyrazole-1-carbothioamide had high binding energy of −128 kcal/mol. The binding pocket of the ligand was made of residues comprising of two chains of the p65 of the DNA binding site and the DNA. Asn 186, Arg187, Pro189, Asp217, Lys218 and Val248 of chain A and Arg246 of chain B along with guanine of the nearest DNA chain forms the pocket. This is consistent with the author’s results that the ligand docks into the cleft adjacent to serine at 276, the important residue for phosphorylation and activation of the protein complex. Our results showed the ligand firmly near the major groove of the DNA. They have also suggested that at concentrations of 100–200 micromoles, the compound was able to decrease the expression of IL-8 and *VCAM1* gene expression in their system [34]. Docking experiments of zerumbone were not so successful in the presence of the DNA. However, docking performed without the DNA involving one of the chains while keeping the same binding area showed that zerumbone forms a hydrogen bond with Arg33. Asn186 is a common residue while other residues involved include Gly31, Gln193 and Lys195.

The Figure 4a shows the position of both the zerumbone and the control. According to Chen *et al.* [36] and Cramer *et al.* [37] the DNA binding region of the human p65 component comprises of residues 33, 35, 36, 38, 39, 41–44, 122–124, 187, 218, 220, 246 and 247. Our docking results showed that the inhibitor bound to residues 187 and 218 while zerumbone bound to residue 33 of the binding site. The other residues were also around the binding site residues. These results suggested that zerumbone could be involved in preventing the p65-DNA binding [36,37].

Takada *et al.* [15] had shown earlier that zerumbone pretreatment inhibits the NF-κB complex indirectly in a time dependent and concentration dependent manner at concentrations of 50 micromoles. Our results appear consistent with their findings because in our case also when no docking poses are obtained for the p65-DNA bound complex. However, in the absence of the DNA, when the p65 has not yet located to the nucleus, zerumbone can bind to it. They also suggest that the pre-treated cells do not induce gene expression. Hence combining all results, we can hypothesize that once zerumbone binds to the p65 component and it translocates to the nucleus, a strong binding to the DNA is prevented hence gene expression is decreased and prevented altogether in a time dependent manner.

A docking experiment with p50 component of the NF-κB bound to DNA (1SVC) was also conducted. Figure 4c,d shows the results. Both the inhibitor as well as zerumbone was able to dock into a pocket near the major groove of the DNA chain. The inhibitor was embedded deeper than zerumbone. The LIGPLOT results indicated that the binding residues common to both involved Pro246, Asn247 and Gln277 and two adenine residues of the DNA. These were also the residues in the binding pocket of zerumbone. The inhibitor had a hydrogen bond with His144 and other residues comprised of Tyr60, Thr146, Lys147, Ser 211, Lys244, Ala245 and Pro 246. According to Chen *et al.* [36] the DNA binding site residues of the p50 subunit comprised of 56, 58, 59, 61, 62, 65–68, 143, 145, 146, 243, 274, 276, 307 and 308. Comparing our results of the binding pocket, we observed that most of the residues of the binding pocket of the control inhibitor, except for Thr146, were adjacent to the suggested residues. The results are consistent with those obtained by Piccagli *et al.* [35] who propose a similar binding pocket in the murine p50 subunit of NF-κB. For zerumbone all binding residues were adjacent to the actual residues [35]. The binding energy obtained for the zerumbone-p50-DNA complex was −65.66 kcal/mol, which was surprisingly higher than the coumarin derivative with −48.81 kcal/mol. These results also indicated affinity of zerumbone for NF-κB complex inhibition. As shown by the experimental results of Takada *et al* [15], zerumbone was able to bind to the p65 and the p50 component of the complex with some affinity. However, they suggested that zerumbone is essentially able to inhibit a step upstream that leads to the activation of NF-κB [14]. Experimental data in our lab [38] also showed that NF-κB gene expression was downregulated in breast cancer cells in the presence of zerumbone with a mean intensity ratio of 0.53. This means that zerumbone probably affects gene expression of the proteins rather than inhibiting the actual protein.

#### 2.2.3. Inhibitor of ΚB Kinase Complex (IKKs)

##### IKKβ

The NF-κB is prevented from translocating to the nucleus by the IκBα [39]. Upon triggering of the TNF signal, another complex in the cytoplasm located upstream from the NF-κB phosphorylates the IκBα. This kinase molecule called the inhibitor of NF-κB kinase (IKK) is a complex of two kinase subunits, IKKα and IKKβ and a noncatalytic subunit, IKKγ, also called NEMO [31,32,40]. The ligand bound structure of IKK beta (3RZF) obtained from PDB database was obtained for docking studies. The results showed that (4-{[4-(4-chlorophenyl)pyrimidin-2-yl]amino}phenyl)[4-(2-hydroxyethyl)piperazin-1-yl]methanone is bound in the pocket between the beta sheets of the *N*-terminal lined by residues involving the kinase domain (KD) [31]. The strong binding of the ligand in the conserved D166-L167-G168 triad is often used for inhibition of protein kinases. Our results showed that the X-ray crystal inhibitor had high binding energy of −140.5 kcal/mol which is consistent with the experimentally reported results of a strong kinase inhibitor [31]. It showed complete inhibition of the anti-inflammatory activity at 100 micromoles/kg of the model animal. Although the binding occurred in the conserved ATP binding pocket that is essential to activate the kinase, the triad was not involved. Docking results given in Figure 5a,b show that zerumbone molecule is also held in the ATP active pocket by nonbonded interactions. Thus no visible hydrogen bonds can be seen even with a binding energy of −64 kcal/mol.

The IKKα and IKKβ subunits are activated by phosphorylation of two serine residues (Ser177 and Ser181 for IKKβ, and Ser176 and Ser180 for IKKα located in an activation loop. Both IKKs have three significant regions, the kinase domain (KD) which is the activated due to ATP binding, the ubiquitin domain (UD) and the scaffold dimerization domain (SDD). In the canonical pathway, NF-κB is activated by the IKK beta subunit and IKKα has little or no role to play [31]. Several studies have been published that target IKKβ [41,42,43,44,45].

The ATP binding site is a narrow, hydrophobic pocket whose floor is formed by a *C*-terminal β-sheet, and its roof is formed by another highly conserved glycine rich loop of the beta sheet 1 of the *N*-terminal. This loop serves to fasten the χphosphate of ATP and a regulatory flap above the ATP binding site [41]. The article by Kalia and Kukol [32] reported that the potential inhibitors should have several hydrophobic centers that fitted deep into the ATP pocket and made interactions with residues especially Asp145/Asp146 and Lys147/Lys44. The crystallized inhibitor proposed by Xu *et al.* [31] met the requirements of being highly hydrophobic with several aromatic rings with some potential proton donors and acceptors which showed a binding energy of −140.5 kcal/mol. Although it did not have any interaction with the suggested key residues, the most probable reason for its high binding could be the formation of hydrogen bonds between the Arg20, Arg31 and Lys106 (Figure 5b). While zerumbone on the other hand is a small structure with only one active carbonyl group (Figure 5a,b). The structure is hydrophobic fitting into the KD and may prevent the ATP binding.

##### IKKα (human) with Zerumbone

Since the crystal structure for human IKK was not available in the PDB Database, the homology model of model of hIKKα was first predicted and then used for further experiments. We modeled amino acid residues between 16 and 300 of the IKKβ which include the binding site for our experiment. The modeled structure of hIKKα showed 63% sequence identity with the catalytic region of the template IKKα (3RZF) of *Xenopus laevis*. The structure obtained illustrated that 86.5% of bond lengths and 70.4% of bond angles were within limits. Seventy-two point seven percent of modeled residues were found in the core region. Figure 6a shows the Modeller v 9.12 generated alignment [46]. According to Xu *et al.* [31], the kinases IKKα and IKKβ fold like a pair of shears with the amino acid residues 1–394 forming the handles of the shear.

The alignment showed the identical KD residues between the template xIKKβ and the model hIKKα with asterix below them. From the alignment, it could be seen that the kinase domain (KD) of the two proteins were almost superimposed. The RMSD value estimated using Swiss-PDB Viewer of the model hIKKα from the template is 0.47 Å [47]. The modeled structure of the catalytic site of the protein is presented in Figure 6c.

**Figure 7:**
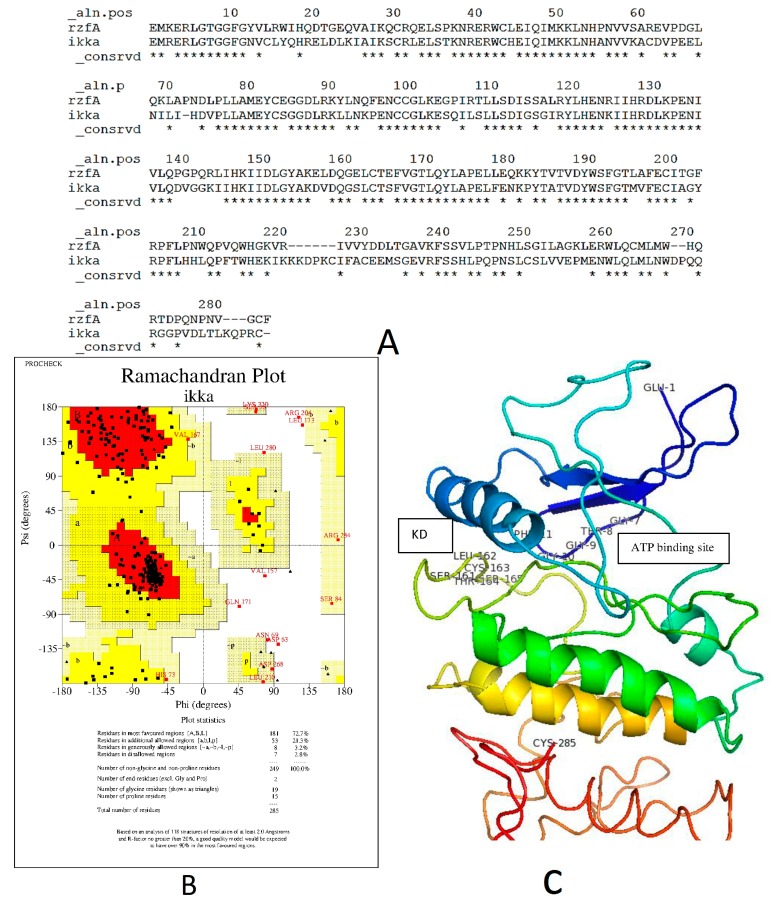
The predicted structure of hIKKα. (**A**) The Modeller9.12 generated alignment of catalytic region of IKKα and IKKα. Residues that are common among both protein sequences are labelled as * below each residue column; (**B**) Ramachandran plot for the predicted protein; (**C**) The complete model of the predicted structure.

The pair-wise alignment (Figure 7a) showed that residues from E16–G27 (E1–G11) were the same. Numbers in parenthesis are written from the alignment generated. This is the region with the glycine rich motif G_22(7)_TGGFG_27(11)_ that forms part of the ATP binding site that holds the χphosphate of the ATP during activation of the kinases. The activation loop of the KD with residues from 176–180 (162–166 in the Figure 7a) consisted of Ser176(161)-Leu177(162)-Cys178(163)-Thr179(164)-Ser180(165). This is a conserved loop because of the Ser176 (161) and Ser180 (165) that are supposed to be phosphorylated for activated kinase. The catalytic loop of the hIKKα is comparable to the similar region of the xIKKβ consisting of residues I_164(150)_DLGYA_169(155)_. This region contains the DLG triad that is deemed necessary for all kinase activity. The IKKα lacks the ubiquitin like domain that is present in the IKKβ and was not modeled.

The docking of zerumbone to IKKα in the ATP binding site showed binding energy of −33.51 kcal/mol. The binding pocket consisted of Glu4(19)-Leu6(21)-Gly85(101)-Lys90(106). The molecule was held by hydrophobic interactions (Figure 7b). The known ligand of xIKKβ showed a strong binding of −155.5 kcal/mol in the pocket of IKKα. Two of the binding residue positions were same as the IKKα with Arg31-Lys106 overlapping with IKKβ at Leu16(31)-Lys90(106).

Our docking results highlight that zerumbone can possibly inhibit kinases in the NF-κB signaling pathway, which was consistent with the results of Takada *et al.* [15] and Weng *et al.* [48] where they have pointed out that zerumbone cannot directly inhibit the NF-κB but a step in the signaling pathway is inhibited that prevents the activation of NF-κB.

In summary, we can propose that the anticancer effect of zerumbone can occur by inhibiting the effect of NF-κB. Thus, zerumbone has two targets along the TNF pathway, the IKKβ and the TNF, for inhibition with equal binding energy. According to the published data by Takada *et al.* [15] the zerumbone mediated inhibition followed by the NF-κB non-canonical pathway with NIK as an intermediate molecule. The most probable target was the IKKβ which also confirmed experimentally by Takada *et al.* [15]. Weng *et al.* [48] have also proposed IKKα as an additional target. They propose that 50 micromoles of zerumbone could decrease the phosphorylation of Akt but it could be restored by addition of wild type IKKα into the cell culture. So IKKα was a step upstream of Akt that could be targeted [48]. Nevertheless, these results require further investigation.

## 3. Experimental Section

### 3.1. Protein and Ligand Starting Structures 

Three dimensional structures of the proteins were obtained from Protein Data Bank [49]. Details are given in Table 1. Preference was given to the selection of ligand bound structures since they already have identified active sites with bound ligands. The structure of zerumbone was obtained from Pubchem database (structure ID: 5470187). For structures without X-ray inhibitors, molecules with known activity against the protein were used.

**Table 1:** A summary of proteins’ name from the tumour necrosis factor (TNF) pathway and their Protein Data Bank (PDB) codes used in this study.

Pathways	Molecule (PDB ID)
Tumour necrosis factor	Tumor necrosis factor (2AZ5)
	IKKβ (3RZF)
	IKKα (Predicted)
	Nuclear factor κβ (p50) (1SVC)
	Nuclear factor κβ RelA (p65) (2RAM)

### 3.2. Homology Modeling

The crystal structure for human inhibitor of κβ kinase kinase α (IKKα) subunit is not available in the Protein Data Bank (University of California, San Diego, CA, USA). Using the structure of IKKα (3RZF.pdb) the structure of the catalytic site of IKKα was predicted by homology modeling using Modeller 9.12 (Eswar and Webb, 2006; available on line: http://salilab.org/modeller). The quality of the structures was checked with PROCHECK [50]. Similarly, the structure of cystine rich domain of the human Frizzled protein FZD8 was predicted using 4F0A.pdb as the template [51].

### 3.3. Preparation of Structures for Docking

Discovery Studio 2.5.5 (Accelrys, Inc., San Diego, CA, USA) was used to prepare the protein and ligand as inputs based on CDOCKER protocol (Accelrys, Inc.). Ligands that were bound to the proteins in the crystal structures were used to compare the results. Some ligands reported as inhibitors in the literature were also used for control docking to compare with zerumbone. CDOCKER adds and minimizes the energy after adding the polar hydrogens on the protein while keeping the heavy atoms fixed. All-atom representation was applied to assign formal and partial charges to the ligand. The formal charges were assigned to match the protonation state of the atoms at pH 7, while the partial charges were based on Momany-Rone force field [52]. The binding site was detected from the coordinates of the original ligand of the PDB file or by using the *find sites from receptor cavities* in the Tools section under Define and Edit Binding Sites in the Discovery Studio.

### 3.4. Docking with CDOCKER

The docked poses were ranked according the lowest CDOCKER energy which is calculated based on the internal ligand strain energy and the receptor-ligand interaction energy and the lowest CDOCKER Interaction Energy, which is a measure of the nonbonded interactions between the ligand and protein. All docked complexes were further used to calculate the binding energies using the *Calculate Binding Energies* protocol in Discovery Studio. The binding energies are calculated based on the following general equation:

(1) $$E_{binding}=E_{complex}-(E_{protein}-E_{ligand})\;where\;E\;is\;the\;energy$$

CDOCKER program uses the CHARMM force-field energy calculations represented by the equation:

(2) $$V=\underset{bonds}{\sum }k_{b}{\left(b-b_{o}\right)}^{2}+\underset{angles}{\sum }k_{\theta }{\left(\theta -\theta_{o}\right)}^{2}+\underset{dihedrals}{\sum }k_{\phi }[1+cos(n\phi -\delta )\;+\underset{impropers}{\sum }k_{\omega }{\left(\omega -\omega_{o}\right)}^{2}+\underset{Urey-Bradley}{\sum }k_{u}{\left(u-u_{o}\right)}^{2}\;+\underset{nonbonded}{\sum }\epsilon \left[{\left(R_{{min}_{i,j}}/r_{i,j}\right)}^{12}-{\left(R_{{min}_{i,j}}/r_{i,j}\right)}^{6}\right]+\frac{q_{i}q_{j}}{\epsilon r_{ij}}$$

where *k_b_* is the bond force constant and *b* − *b*_0_ is the distance from equilibrium that the atom has moved. The second term in the equation accounts for the bond angles where *k*_θ_ is the angle force constant and θ − θ_0_ is the angle from equilibrium between 3 bonded atoms. The third term is for the dihedrals where *k*_φ_ is the dihedral force constant, *n* is the multiplicity of the function, φ is the dihedral angle and δ is the phase shift. The fourth term accounts for the impropers, that is out of plane bending, where *k*_ω_ is the force constant and ω − ω_0_ is the out of plane angle. The Urey-Bradley component (cross-term accounting for angle bending using 1,3 nonbonded interactions) comprises the fifth term, where *k_u_* is the respective force constant and *u* is the distance between the 1,3 atoms in the harmonic potential. Nonbonded interactions between pairs of atoms (*i*, *j*) are represented by the last two terms. By definition, the nonbonded forces are only applied to atom pairs separated by at least three bonds. The van Der Waals (VDW) energy is calculated with a standard 12-6 Lennard-Jones potential and the electrostatic energy with a Coulombic potential. In the Lennard-Jones potential above, the *R*_min*i*,*j*_ term is not the minimum of the potential, but rather where the Lennard-Jones potential crosses the *x*-axis [53]. 

Pathway in Figure 1 was drawn using Pathvisio beta 3.0 software [54] and Pymol was used to visualize the docked ligands [55].

## 4. Conclusions 

Zerumbone exhibits the high binding affinity from molecular docking studies with several key signal proteins (Tumour necrosis factor, Nuclear factor κB, ΚB kinase) in the tumor necrosis factor pathway indicating its favorable binding not only at one target proteins. The key binding residues in correlated with the experimental studies have been identified for each target. Since TNF can work at pro-apoptotosis and anti-apoptosis, the action of zerumbone is most probably on the anti-apoptotic pathway, where it inhibits the kinases as indicated by the strong CDOCKER interaction energy and can involve in the activation of NF-κB. Once NF-κB is inhibited from translocating to the nucleus, the pro-apoptotic action of TNF can proceed.
